# Lipid Mediators in Host–Pathogen Interactions during *Leishmania* Infection: Friends or Foes?

**DOI:** 10.1021/acsinfecdis.6c00200

**Published:** 2026-05-25

**Authors:** Yasmin Monara Ferreira de Sousa Andrade, Darlaine Alves Silva, Tainá Larissa Pires Nascimento, Matheus Oliveira Sousa, Beatriz Lorrany Araújo Carvalho, Jonilson Berlink Lima, Valéria M. Borges, Théo Araújo-Santos

**Affiliations:** † Núcleo de Estudos de Agentes Infecciosos e Vetores (NAIVE), Centro das Ciências Biológicas e da Saúde, 423876 Universidade Federal do Oeste da Bahia (UFOB), Barreiras 47808-021, Bahia, Brasil; ‡ Laboratory of Inflammation and Biomarkers, Gonçalo Moniz Institut, Oswaldo Cruz Foundation, Salvador 40296-710, Brazil

**Keywords:** lipid mediator, *Leishmania*, host−pathogen interaction, eicosanoids

## Abstract

Lipid mediators can control the inflammatory response in infectious diseases, including leishmaniasis. However, these mediators may promote antagonistic roles in the *Leishmania*–host interaction depending on the species involved in the infection. Herein, we analyzed the role of mediators in the *Leishmania*–host interaction in the experimental and clinical context, aiming to identify the main cell types studied, as well as the main eicosanoids and their influence during the infection. The main lipid mediators studied were the eicosanoids LTB_4_ and PGE_2_, which are related to the inflammatory response in cutaneous and visceral leishmaniasis. In vitro models using macrophages and neutrophils infection reveal that LTB_4_ plays a fundamental role in reducing the parasite load, while PGE_2_ and PGF_2α_ suppress the immune response, favoring the survival of the parasite in the host. In the in vivo infection, PGE_2_ is related to the visceralization process of the disease and the persistence of tegumentary lesions. An emerging role in pathophysiology has been pointed out for the mediators of the HETE class and for Resolvin D_1_, which act favoring *Leishmania* infection and are associated with more severe cases of the disease. Thus, it can be concluded that lipid mediators play crucial roles in the *Leishmania*–host interaction, modulating the inflammatory response and disease progression. Studies exploring the contribution of intervention in the production of lipid mediators during the course of the disease are still needed.

## Graphical abstract

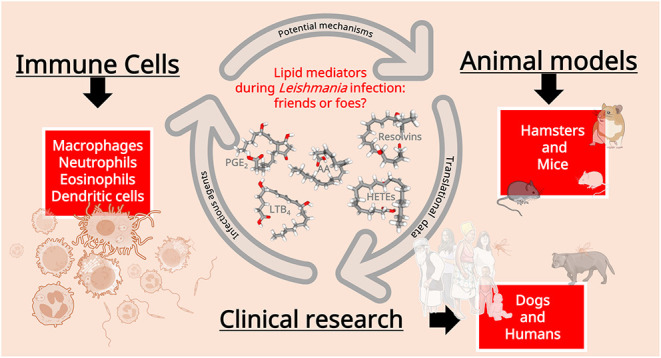

## Introduction

Leishmaniasis is classified as a neglected disease that impacts numerous populations throughout tropical and subtropical regions worldwide.^1^ According to the World Health Organization (WHO), an estimated 700,000 to 1 million new cases occur each year, with approximately 12 million people currently affected.^2^

The etiological agent of leishmaniasis is *Leishmania*, a genus of protozoa belonging to the family Trypanosomatidae.^3^ The clinical outcome of leishmaniasis is strongly influenced by the infecting *Leishmania* species, ranging from cutaneous leishmaniasis (CL), which can present as localized (LCL) or diffuse (DCL) lesions, to the more severe mucocutaneous form (MCL) (Figure 1). Visceral leishmaniasis (VL), the most severe manifestation of the disease, occurs in both humans (HVL) and dogs (CVL) with similar immunopathological features and may also be associated with tegumentary lesions, as observed in postkala-azar dermal leishmaniasis (PKDL).^4^ The clinical outcome of the infection depends on a delicate balance between the parasite and host immune response. Innate and adaptive immune mechanisms play a crucial role in controlling *Leishmania* infection, with the activation of macrophages, neutrophils, dendritic cells, and T lymphocytes as key modulators of disease progression or resolution.^3^

**Figure 1:**
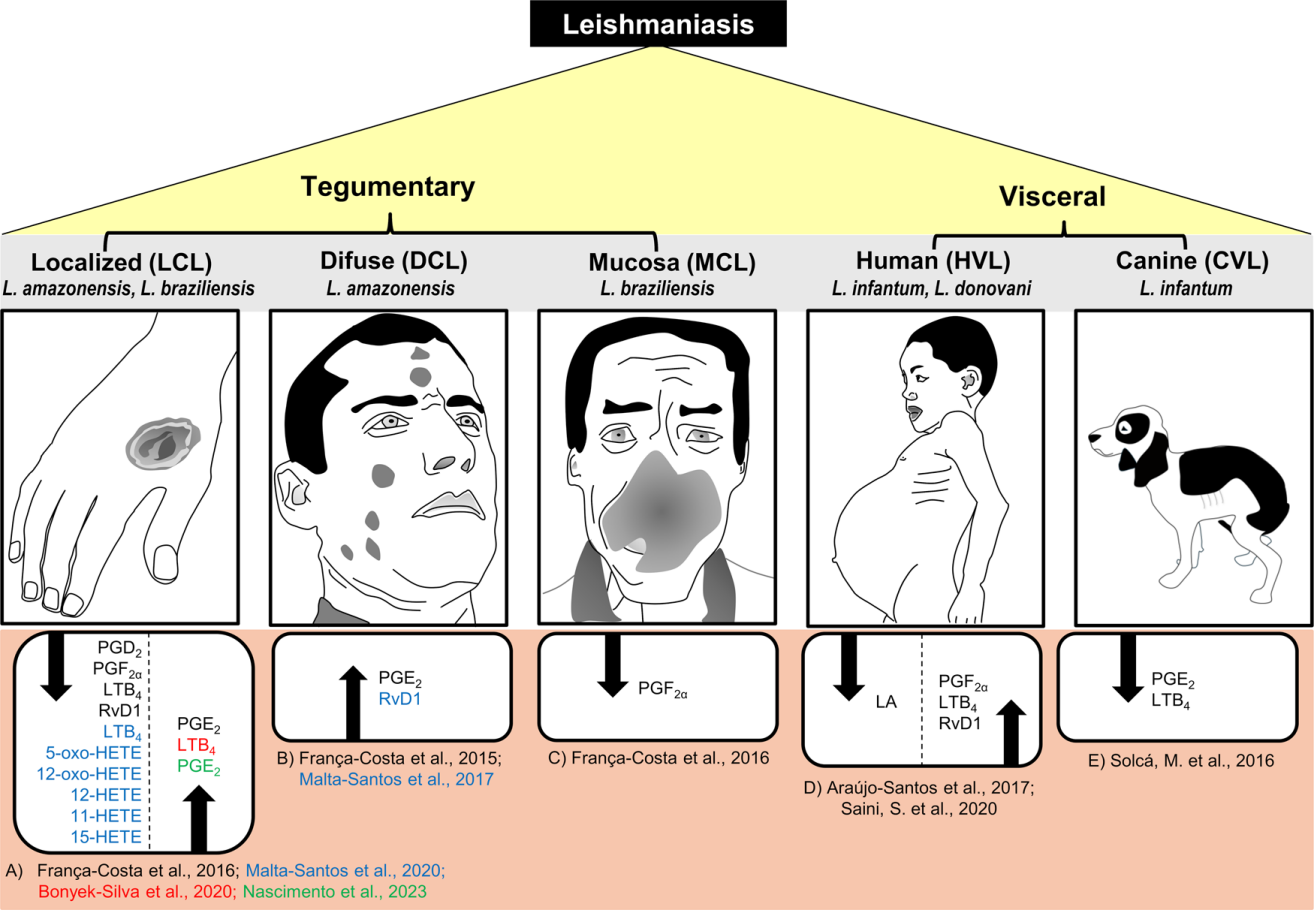
Leishmaniasis presents a clinical spectrum that varies from cutaneous forms (localized, diffuse and mucosal) to visceral forms (in humans and dogs), caused by different species of *Leishmania*. Below each clinical form, the main lipid mediators whose production is increased (arrow pointing up) or decreased (arrow pointing down) are highlighted, as described in the literature. (A) Lipid mediators in black: evaluation of plasma from patients with and without the LCL; in blue: evaluation of plasma from patients cured 60 days after treatment; in red: evaluation of plasma from patients with the LCL, with and without diabetes; and in green: evaluation of lesions from patients with the LCL form versus healthy skin. (B) Lipid mediators in black: evaluation of plasma from patients with and without DCL; and in blue: evaluation of plasma from patients with the DCL compared to patients with the LCL. (C) Evaluation of plasma from patients with and without the MCL. (D) Evaluation of plasma from patients with and without HVL. (E) Evaluation of plasma from dogs with and without CVL. The studies cited reinforce the modulating role of lipid mediators in the inflammatory response and disease progression. Illustrations of the different clinical forms of leishmaniasis by Théo Araújo-Santos.

Parasite-driven activation of immune cells induces production of immunoregulatory mediators, essential in modulating the immune response during leishmaniasis.^5^ Among these immunoregulators, lipid mediators are recognized as major regulators of inflammation. These bioactive lipids are synthesized within specialized cellular organelles known as lipid droplets (LDs)^6^ and are derived from polyunsaturated fatty acids (PUFAs) of the omega-6 family—arachidonic acid (AA)—or omega-3—eicosapentaenoic acid (EPA) and docosahexaenoic acid (DHA).^7,8^

Depending on which PUFA is metabolized, lipid mediators can be classified as either pro-inflammatory or pro-resolving. Pro-inflammatory lipid mediators promote and coordinate the inflammatory response, while pro-resolving mediators facilitate its resolution.^9^ Pro-inflammatory lipid mediators include prostaglandins (PGs), leukotrienes (LTs), and thromboxanes (TXs), whereas specialized pro-resolving mediators (SPMs) comprise lipoxins (LXs), resolvins (Rvs), protectins (PDs), and maresins (MaRs).^8,9^ In this context, it is also important to define eicosanoids, which are lipid mediators derived exclusively from the oxidation of arachidonic acid (AA). This group includes PGs, LTs, TXs, and LXs.^8^

The enzymatic biosynthesis of lipid mediators begins with the deesterification of PUFAs from membrane phospholipids, a process catalyzed by phospholipase A_2_ (PLA_2_) enzyme.^10^ The subsequent steps involve the action of three major enzyme families: cyclooxygenases (COXs), lipoxygenases (LOXs), and cytochrome P450 (CYP450). These enzymes initiate a cascade of specific pathways and enzymatic reactions that lead to the production of bioactive lipid mediators.^10^

An imbalance in the synthesis of different eicosanoids can contribute to parasite persistence by promoting an anti-inflammatory environment or, conversely, enhance host resistance by stimulating pro-inflammatory pathways.^5,11^ Gaining a deeper understanding of the intricate role of eicosanoids and their synthesis within lipid bodies in leishmaniasis is essential for developing novel therapeutic strategies aimed at host-directed immune modulation. This literature review provides a comprehensive analysis of the role of lipid mediators in the various clinical forms of leishmaniasis, focusing on defining their profiles and elucidating their contribution to disease pathogenesis and progression.

### Lipid Mediators Roles during *Leishmania* Infection of Immune Cells

Different cell types can be infected by various *Leishmania* species, and these cells play pivotal roles in the immune response during Leishmaniasis. Therefore, evaluating the behavior of these cells upon exposure to *Leishmania* infection is essential and has been extensively studied in vitro. These studies contribute to a deeper understanding of the cellular interactions and immune mechanisms involved in the pathogenesis of the disease.

In this context, lipid mediators contribute to the initial immune response to *Leishmania*, since these molecules control both the cell recruitment and activation at the infection site.^5^ In the early steps of the infection, the parasites are inoculated by sand fly bite in the host together with *Lutzomyia longipalpis* saliva, which contains chemoattractant proteins that rapidly recruit neutrophils and monocytes to the infection site,^12–14^ even the artificial needle injection of the parasites also induces the recruitment of these cells.^15–17^ Once there, neutrophils activate their microbicidal mechanisms, including phagocytosis and the release of extracellular traps (NETs).^18,19^ However, the influence of neutrophils on the outcome of leishmaniasis varies depending on the *Leishmania* species. Human and murine neutrophils infected by *Leishmania infantum* in the presence of *Lutzomyia longipalpis* salivary gland sonicate (SGS) increase PGE_2_ production, promoting parasite persistence.^20,21^ PGE_2_ stimulated by sand fly saliva can control the parasite killing of *L. infantum* inside monocytes and neutrophils by inhibiting the antiparasitic effects of LTB_4_.^22^ Similarly, LTB_4_ release by neutrophils is essential for controlling *Leishmani amazonensis*, as it promotes cell degranulation and the release of reactive oxygen species.^23^ Later studies demonstrated that this effect is mediated by fibronectin-stimulated neutrophil degranulation, which enhances LTB_4_ production by infected macrophages.^24^ Moreover, neutrophils infected with *Leishmania major* exhibit elevated levels of LTB_4_ and reduced levels of lipoxin A_4_ (LXA_4_), suggesting a lipid mediator profile that favors a pro-inflammatory and potentially leishmanicidal response.^25^ Moreover, neutrophil phagocytosis modulates infection in a manner dependent on the type of cell death: apoptotic neutrophils increase parasite burden (via TGF-β1 and PGE_2_), whereas necrotic neutrophils induce leishmanicidal activity (dependent on TNF-α and neutrophil elastase).^26^ Thus, the neutrophil role in *Leishmania* infection depends on the species involved and which lipid mediators are present in the context of the infection.

Monocytes play a crucial role in pathogen defense and are rapidly recruited to the site of infection.^27,28^ Studies indicate that Ly6C+ monocytes, which express the granulocyte marker GR1, migrate from the bloodstream to lesions and control parasites through the release of reactive oxygen species (ROS), contributing to resistance against *L. major* infection.^14,19,29^ In contrast, human monocytes were infected with *L. amazonensis* exhibit altered adhesion to connective tissues, facilitating parasite dissemination and lesion progression in leishmaniasis.^30^ The influence of lipid mediators on *Leishmania*-infected monocyte cultures remains poorly understood. However, the cyclooxygenase pathway has been linked to *Trypanosoma cruzi* invasion in an in vitro model, where human monocytes treated with aspirin significantly internalized reduced trypomastigote forms.^31^

Eosinophils are multifunctional leukocytes that play a key role in host defense against helminth infections.^32^ Additionally, they contribute to allergic processes, local immunity, and tissue repair.^33,34^ When activated, eosinophils release various proteins stored in their granules^35^ and secrete high levels of phospholipase A_2_, distinguishing them from other leukocytes in this regard.^36^ This enzyme enhances phospholipid hydrolysis and the production of lipid mediators,^37,38^ such as prostaglandins, leukotrienes, 5-oxo-ETE, lipoxins, protectins, and resolvins, which regulate various eosinophil functions.^33^ During *Leishmania* infections, eosinophils are recruited to the inoculation site of *L. amazonensis*, *Leishmania mexicana*, and *Leishmania donovani*.^39–42^ However, the relationship between eosinophil infiltration and reduced parasite burden varies depending on the *Leishmania* species involved.^39,43,44^ Eosinophils enhance macrophage capacity to control intracellular *L. amazonensis* infection in vitro by secreting PGD_2_, which regulates macrophage activity, making the eosinophil/PGD2 axis a potential adjunct therapeutic strategy for diseases caused by *L. amazonensis*.^45^

Dendritic cells (DCs) are professional antigen-presenting cells (APCs) that, upon encountering inflammatory or infectious stimuli, undergo maturation and migrate to T-cell zones within lymphoid organs, thereby serving as a critical link between innate and adaptive immunity.^46,47^ The various DC subtypes produce lipid mediators that modulate their immune functions, such as LTs and PGE_2_, which promote DC maturation and T-cell activation, enhancing the adaptive immune response. In contrast, PGI_2_ suppresses these functions, contributing to inflammation resolution.^47–49^ Additionally, SPMs also modulate DC maturation and function, influencing T-cell polarization and antibody production by B cells.^50^ *Leishmania* impairs the activation and migration of DCs, which is directly associated with parasite persistence in the host.^51–53^ However, direct contact between PMNs and DCs infected with *L. amazonensis* through the C-type lectin receptor (DC-SIGN on the DC surface) increased PGE_2_ levels and induced ROS, thus contributing to parasite elimination within the DCs.^54^

Macrophages are essential cells involved in the development, tissue homeostasis, repair, and immune defense. They act in innate immunity as phagocytes and in adaptive immunity by presenting antigens to T cells, thereby initiating specific immune responses.^19,55^ In addition, they modulate the synthesis of reactive oxygen species (ROS) and produce inflammatory mediators including bioactive lipids. Macrophages are the primary host cells for *Leishmania*, playing a crucial role in the survival, replication, and differentiation of the parasite within the host.^19,56^

*L. amazonensis* is one of the species responsible for tegumentary leishmaniasis, and in vitro studies with macrophages infected by this parasite have demonstrated that different immunometabolic mechanisms can both promote or limit its replication. Among the mechanisms involved in protozoan elimination, it is known that the activation of the purinergic P2X7 receptor stimulates the production of LTB4 ^57^ and cysteinyl–leukotrienes (Cys-LTs), including LTC_4_ and LTD_4_,^58^ which in turn reduce the *L. amazonensis* parasite load in cultured macrophages. The P2X7/LTB_4_ axis contributes to parasite clearance by activating the NLRP3 inflammasome, leading to reactive oxygen species (ROS) production, and IL-1β secretion.^59^ Furthermore, modulation of the LTB_4_ pathway directly regulates the host resistance or susceptibility to infection.^60^ Additionally, inhibition of arginase I or ornithine decarboxylase (ODC) has been shown to block *L. amazonensis* replication, indicating that arginine metabolism is crucial for parasite survival.^61^

On the other hand, the activity of phospholipase A_2_ (PLA_2_) promotes suppression of IL-2 and induction of PGE_2_, potentially acting as a progression factor for cutaneous leishmaniasis (CL).^62^ Within lipid regulation, macrophage autophagy has also been described as promoting infection by modulating PGE_2_ production and creating an environment favorable to parasite replication.^63^ Additionally, macrophages previously infected with HIV-1 exhibited a higher parasite burden after *Leishmania* infection, suggesting a pathogenic cooperation between the two microorganisms.^64^

During in vitro studies of macrophage infection by *L. mexicana* and *L. braziliensis*, various immunometabolic mechanisms have also been associated with the modulation of susceptibility or control of the infection, with emphasis on the eicosanoids PGE_2_ and LTB_4_. Among the findings demonstrating a leishmanicidal effect, infection with *L. braziliensis* was shown to induce LTB_4_ production.^65^ Supporting this effect, inhibition of PGE_2_ biosynthesis resulted in reduced inflammation and decreased parasite burden in macrophages infected with *L. braziliensis*, indicating that suppression of the PGE_2_ pathway may favor parasite elimination.^66^ In a *L. mexicana* infection model, activation of the nuclear receptor PPARγ led to the inhibition of the cPLA2-COX-2 pathway and an increase in reactive oxygen species (ROS), PGE_2_, and PGF_2α_, culminating in restriction of parasite replication.^67^ Despite the increased PGE_2_ in this context, the combined effect with ROS and PGF_2α_ suggests that PPARγ-induced oxidative mechanisms may override the permissive effects of PGE_2_.^67^ Conversely, macrophages derived from patients with CL and diabetes mellitus (CL+DM) exhibited an eicosanoid profile with a relative increase in PGE_2_ compared to LTB_4_, favoring parasite replication.^68^ These data reinforce the critical role of eicosanoids in the immunoregulation of tegumentary leishmaniasis, highlighting the importance of the balance between PGE_2_ and LTB_4_ as a pivotal mechanism in the outcome of the host–parasite interaction.

Lipid mediators also play crucial roles in regulating the immune response against VL. PGE_2_, for example, has context-dependent effects and can contribute to parasite elimination by promoting nitric oxide (NO) production by activated macrophages.^5^ However, most studies indicate that PGE_2_ favors parasite replication through activation of the COX-2 pathway.^69^ Increased secretion of PGE_2_ is also associated with the activation of intracellular signaling pathways such as protein kinase C α (PKC-α), reinforcing the idea of a cellular mechanism that supports parasite survival.^70^ Furthermore, activation of the PGE_2_/EP2 pathway has been shown to induce expression of the transcription factor NRF2, promoting parasite persistence within macrophages.^71^ Consistently, elevated levels of PGE_2_ have also been correlated with higher infection rates,^72^ as well as with the downregulation of MHC class II antigen expression.^73^ The immunomodulatory role of lipids is also demonstrated by the ability of parasite-derived lipids to induce an M2 macrophage phenotype, which is associated with the production of SPMs such as RvD_2_, PDx, and 7-MaR1, and an increase in infection.^74^ Supporting this immunosuppressive bias, macrophages from malnourished BALB/c mice exhibit higher production of PGE_2_, contributing to immunosuppression and parasite persistence.^75^

Other studies point to the involvement of specific receptors in mediating these lipid effects. For example, blocking the FP receptor significantly reduces infection, suggesting that the PGF_2α_/FP pathway favors parasite survival.^76^ Additionally, the release of cPLA_2_ and PGE_2_ in *L. infantum*-infected cells depends on activation of Toll-like receptor 2 (TLR2), reinforcing the role of innate receptors in the lipid response to infection.^77^ The LPG also stimulates PGE_2_ production via TLR1/2 and PPAR-γ activation, further expanding *Leishmania* capacity to manipulate host lipid metabolism.^78^

Other lipid mediators also participate in the dynamics of infection. For example, suppression of LTA_4_H and the DC-SIGN receptor leads to reduced levels of LTB_4_ and IL-1β, thereby favoring infection.^79^ On the other hand, the 15-deoxy-Δ12,14-prostaglandin J_2_ (15d-PGJ_2_) has proven effective in eliminating amastigotes, suggesting therapeutic potential in modulating specific lipid pathways.^80^ Additionally, compounds such as glycyrrhizic acid (GA) exhibit immunomodulatory and leishmanicidal properties,^81^ representing promising therapeutic tools.

Finally, the role of PUFAs should also be highlighted. They increase the production of epoxyeicosatrienoic acids (EETs) and hydroxyeicosatetraenoic acids (HETEs) from AA, as well as increase hydroxydocosahexaenoic acids (HDoHEs) from DHA, these mediators being associated with increased infectivity of *L. infantum* in murine macrophages.^82^ In contrast, linoleic acid (LA), an omega-6 PUFA, specifically acts through the 5-lipoxygenase (5-LO) pathway, promoting a protective response in macrophages against infection by *L. donovani*.^83^

Splenic cells, including resident splenic leukocytes as well as T and B cells, play crucial roles in the immune response against infections in general.^84^ In the context of *Leishmania* infection, these cells also exhibit distinct behaviors regarding the production of PGE_2_, depending on the species involved. In VL caused by *L. infantum*, splenic leukocytes from dogs with active infection produced low levels of PGE_2_, and the exogenous addition of this mediator reduced parasite burden, underscoring its immunomodulatory role.^85^ In contrast, supernatants from spleen cells of BALB/c mice were infected with *L. donovani* contained elevated levels of PGE_2_ and LTC_4_, while cell extracts revealed increased concentrations of 5-HETE and 12/15-HETE. Notably, inhibition of COX activity in this model restored lymphocyte blastogenesis, indicating that AA metabolites regulate lymphocyte function within the splenic environment.^86^ Similarly, during *L. mexicana* infection, splenic cells from BALB/c and C57BL/6 mice treated with indomethacin (INDO), a COX inhibitor, and stimulated by soluble leishmanial antigen (SLA), exhibited reduced PGE_2_ levels. This reduction favored a Th1 response and contributed to parasite elimination, indicating that inhibition of the PGE_2_ pathway may be beneficial in this model.^19,87^

Extending this perspective beyond splenic leukocytes, T cells have also been implicated in the modulation of PGE_2_ during infection. For instance, T cells from BALB/c mice infected with *L. major* showed increased production of PGE_2_, along with IL-1 and TGF-β, which favored disease progression.^19,88^ Additionally, in phagocytic cells derived from B-1 cells (B-1CDP), also in the context of *L. major* infection, elevated PGE_2_ production was observed. This increase induced the release of IL-10, thereby promoting the infection. The use of COX-2 inhibitors blocked PGE_2_ production and consequently reduced the suppressive effect of IL-10, indicating a potential therapeutic pathway to modulate the infection.^89^

Overall, the production of lipid mediators in response to infection by different *Leishmania* species shares common mechanisms, primarily involving the eicosanoids PGE_2_ and LTB_4_, which play a critical role in balancing parasite persistence and elimination, respectively. However, species-specific differences in lipid-induced response are also observed. These complex interactions highlight the role of immunometabolism in leishmaniasis and underscore the need for personalized therapeutic approaches that consider the specific dynamics of each species and the context of the host cells. Herein, we summarize the signaling pathways related to cell and leishmania interactions with consequences of eicosanoid production and stimulation to cell infection with *Leishmania* (Table 1; Figures 2 and 3).

**Figure 2:**
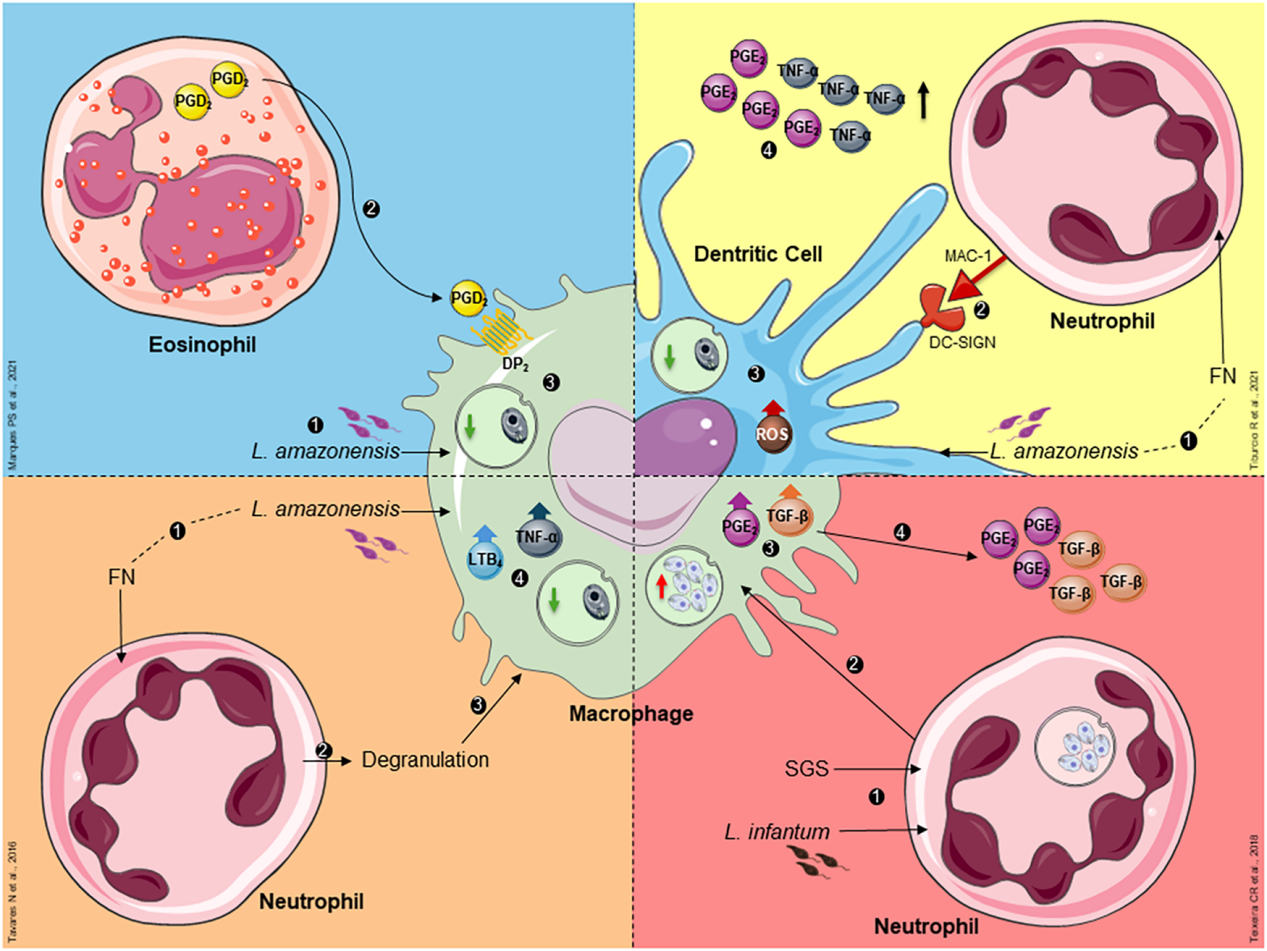
Schematic representation of the interactions between immune system cells, lipid mediators, and *Leishmania* spp. The diagram illustrates how *L. amazonensis* and *L. infantum* modulate the production of lipid mediators in different host cells—including neutrophils, macrophages, and dendritic cells—and how the activity of other immune cells impacts cellular function and parasite survival. Infection regulates the inflammatory response through the production of PGD_2_, PGE_2_, LTB_4_, TNF-α, TGF-β, and ROS, favoring or hindering infection. Furthermore, stimuli FN and SGS participate in modulating parasite–cell interactions. In each quadrant, the numbers 1, 2, 3, and 4 indicate the order of events. Eosinophils (top left): (1) macrophage infection by *L. amazonensis* activates eosinophils; (2) which in turn produces and releases PGD_2_, (3) which interacts with macrophage DP2 receptor, reducing infection; DCs (top right): (1) DC infection by *L. amazonensis* and neutrophil stimulation with FN; (2) infection and stimulation favor the interaction between DC and neutrophil via DC-SIGN and MAC-1 receptors, respectively; (3) the interaction increases ROS synthesis by DC, killing the parasite; (4) release of more PGE_2_ and TNF-α; neutrophils (bottom left): (1) Macrophage infection by *L. amazonensis* and neutrophil stimulation with FN; (2) stimulation promotes neutrophil degranulation; (3) the degranulation drives macrophage production of LTB_4_ and TNF-α, killing the parasite (4); Neutrophils (bottom right): (1) Neutrophil infection by *L. infantum* and stimulation with SGS; (2) neutrophil infection promotes macrophage infection, which in turn produces (3) and releases (4) more PGE_2_ and TGF-β, favoring the anti-inflammatory environment and parasite persistence. PG: prostaglandin; LT: leukotriene; TNF-α: tumor necrosis fator α; TGF-β: transforming growth factor β; ROS: reactive oxygen species; FN: fibronectin; SGS: salivary gland sonicate; DC: dendritc cells.

**Figure 3:**
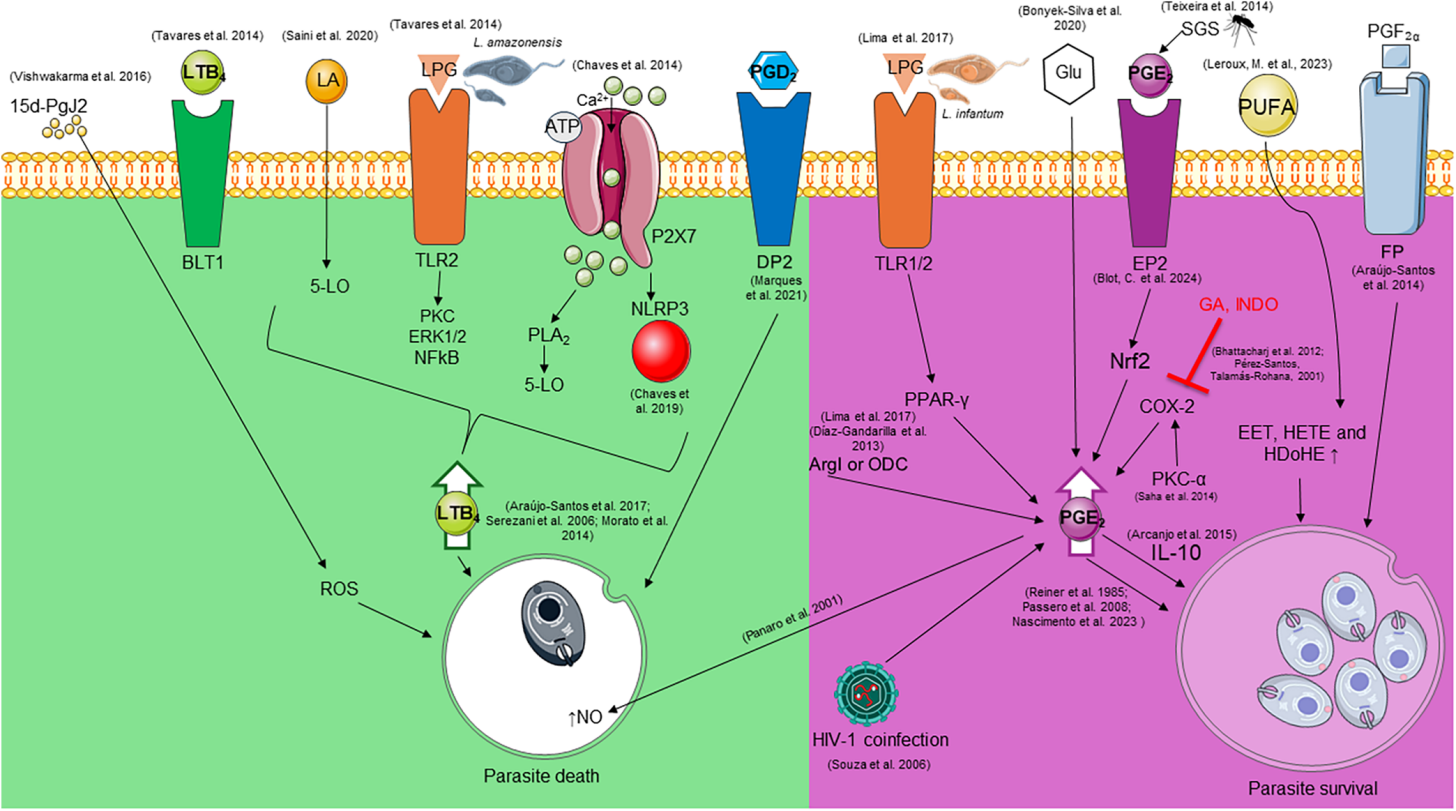
Cellular mechanisms related to lipid mediator production and parasite load during *Leishmania* infection. On the left, in green, there is an increase in LTB_4_ via 5-LO, triggered by stimulation with LA, LTB_4_, PGD_2_, 15d-PGJ_2_, and LPG, which activate different receptors (BLT1, TLR2, P2X7, and DP2), intracellular pathways PKC, ERK1/2, NFκB, and the NLRP3 inflammasome, inducing ROS and NO and leading to parasite death. In contrast, the purple side highlights the increase in PGE_2_ via COX-2 due to the activation of receptors such as TLR1/2, EP2, FP, among others not shown, stimulated by LPG, glucose, SGS, and PGF_2__α_, which activate regulatory pathways including Nrf2, PKC-α, PPAR-γ, and ODC. Together with IL-10, and in cases of coinfection with HIV, these mechanisms favor parasite survival. PG: Prostaglandin; LT: leukotriene; 5-LO: 5-lipoxigenase; LA: linoleic acid; ROS: reactive oxygen species; SGS: salivary gland sonicate; LPG: lipophosphoglycan; NO: nitric oxide; ODC: ornithine decarboxylase; COX: cyclooxygenase; NLRP3: NOD-, LRR- and pyrin domain-containing protein 3; PKC: Protein kinase C; ERK: extracellular signal-regulated kinases; NFκB: Nuclear factor-κB; TLR: Toll-like receptor; FP: prostaglandin F_2__α_ receptor; EP: prostaglandin E receptor; BLT: leukotriene B receptors; DP: prostaglandin D receptor.

**Table 1:** Lipid Mediators during *Leishmania* Infection in Different Cell Types and Experimental Conditions In Vitro and Ex Vivo^a^

species	cell types	organism	stimuli	lipid mediators measured	parasite load	effect	refs	DOI
*Leishmania infatum*	macrophage	human	PGE_2_ and LPS	PGE_2_ ↑	↓	PGE_2_ promotes parasite killing via NO production	^5^	10.1007/s10238-001-8025-0
	J774 macrophage	murine cell line	AA and DHA	EET, HETE, and HDoHE	↑	PUFA increases lipid mediators and enhances parasite infectivity	^82^	10.1002/lipd.12365
	macrophage	human	siRNA targeting LTA_4_H + DC-SIGN	LTB_4_ ↓	↑	silencing LTA_4_H and DC-SIGN reduces IL-1β and favors infection	^79^	10.1016/j.immuni.2013.04.010
	BMDM	C57BL/6 mice	LPG	PGE_2_ ↑	-	LPG increases PGE_2_ viaTLR-1/2 and PPAR-γ	^78^	10.1038/s41598-017-14229-8
	BMDM	C57BL/6 mice	oxylipins isolated from metacyclic promastigotes	↑: 9- and 13-HODE; 5-, 8-, 12- and 15-HETE; 8(9)-EpETrE; 14- and 17-HDoHE; RvD_2_; PDx; 7-MaR1	-	lipids from parasite induce the M2 phenotype, SPMs - and infection	^74^	10.1194/jlr.M091736
	BMDM	BALB/c mice	AA, aspirin	PGF_2α_ ↑	↑	FP receptor blockade reduces infection, indicating a pro-parasitic role of the PGF_2α_/FP pathway	^76^	10.1093/infdis/jiu299
	peritoneal macrophages and macrophages	C57BL/6 mice and human, respectively	PGE_2_ and inhibitors to assess parasite growth in BMDMs	PGE_2_ ↑	↑	PGE_2_/EP2 signaling activates Nrf2 and facilitates infection	^71^	10.1016/j.celrep.2024.114720
	coculture: neutrophils and macrophages	human	*Lutzomyia longipalpis* SGS	PGE_2_ ↑	↑	*L. longipalpis* SGS increases PGE_2_, TGF-β and enhances infection	^20^	10.3389/fmicb.2018.00881
	splenic leukocytes	dogs with and without VL	PGE_2_, PGE_2_ receptors agonists, COX-2 inhibitor	PGE_2_ ↓	↓	cells from dogs with VL have low PGE_2_ and exogenous PGE_2_ reduces infection	^85^	10.1111/pim.12713
	peritoneal macrophages	BALB/c mice	GA	PGE_2_ ↓	↓	GA has immunomodulatory and leishmanicidal properties	^81^	10.1093/jac/dks159
	peritoneal macrophages	BALB/c mice	TLR ligands	PGE_2_ ↑	↑	cPLA_2_ and PGE_2_ release in infected cells depends on TLR2	^77^	10.1128/IAI.00528-16
	peritoneal macrophages	BALB/c mice	LPS, calcium ionophore, COX inhibitors	PGE_2_↑, LTB_4_ ↓	-	malnourished BALB/c macrophages exhibit elevated PGs production, contributing to immunosuppression	^75^	10.1016/j.plefa.2009.04.011
	peritoneal macrophages	BALB/c mice	LPS	PGE_2_ ↑, PGF_2α_ ↑	↑	increased PGE_2_ synthesis correlates with infection rate	^72^	10.4049/jimmunol.134.1.556
	J774 macrophage	murine cell line	15d-PGJ_2_	-	↓	15d-PGJ_2_ effectively eliminates intramacrophage amastigotes	^80^	10.1007/s00109-016-1384-5
	RAW 264.7 macrophage	murine cell line	LPS and IFN-γ	PGE_2_ ↑	-	increase in PGE_2_ secretion occurs via PKC-α	^70^	10.4049/jimmunol.165.7.3985
	peritoneal macrophages	BALB/c mice	PGE_2_, COX-2 inhibitor	PGE_2_ ↑	↑	Leishmania induces COX-2/PGE_2_, promoting parasite survival		10.4049/jimmunol.1400399
	J774 macrophage	murine cell line	AA, LA, COX-2 and 5-LO inhibitors	-	↓	protective response of LA was mediated via 5-LO pathway	^83^	10.1016/j.biochi.2020.04.024
	neutrophil	C57BL/6 mice and human	*Lutzomyia longipalpis* SGS	PGE_2_ ↑	↑	SGS promoted neutrophil apoptosis and PGE_2_ release, enhancing *Leishmania* survival	^21^	10.1189/jlb.0211105
*Leishmania amazonensis*	coculture: eosinophils and macrophages	BALB/c mice	PGD_2_ or DK-PGD2, DP2 receptor antagonist and agonist	PGD_2_ ↑	↓	eosinophil-derived PGD2 controls infection via DP2 activatio	^45^	10.1016/j.cellimm.2021.104316
	macrophage	human	arginase and ODC inhibitors	PGE_2_ ↓	↓	arginase I or ODC inhibition blocks *L. amazonensis* replication	^61^	10.1093/infdis/jiu455
	peritoneal macrophages	BALB/c mice	PLA_2_	PGE_2_ ↑	-	PLA_2_ suppresses IL-2 and induces PGE_2_, may be a progression factor for CL	^62^	10.1007/s00436-007-0871-6
	peritoneal macrophages	BALB/c mice	PGE_2_, indomethacin	PGE_2_ ↑	↑	macrophage autophagy promotes infection by modulating PGE_2_	^63^	10.1016/j.micinf.2008.11.006
	peritoneal macrophages	BALB/c, C57BL/6, SV129, P2X7 receptor–deficient and 5-LO–deficient mice	LTB_4_, ATP	LTB_4_ ↑	↓	P2X7 induces LTB4, essential for *L. amazonensis* elimination	^57^	10.4049/jimmunol.1301058
	peritoneal macrophages	C57BL/6 and knockout mice for P2X7, NLRP3, ASC, Casp-11, and IL1R	LTB_4_, ATP, IL-1β	LTB_4_ ↑	↓	parasite elimination via P2X7/LTB_4_ depends on NLRP3, ROS and IL-1β	^59^	10.1371/journal.ppat.1007887
	neutrophils	human	LPG, zileuton, FLAP inhibitor, BLT1 antagonist	LTB_4_ ↑	↓	LTB_4_ enhances neutrophil-mediated parasite killing via BLT1	^23^	10.1093/infdis/jiu158
	coculture: neutrophils and macrophages	human	fibronectin, zileuton	LTB_4_ ↑	↓	LTB_4_ mediates *L. amazonensis* killing postneutrophil degranulation	^24^	10.4049/jimmunol.1502224
	peritoneal macrophages	BALB/c and C3H/HePAS mice	LTB_4_, BLT1 antagonist, FLAP inhibitor	LTB_4_ ↑	↓	LTB_4_ regulates resistance and susceptibility to *Leishmania* infection	^60^	10.4049/jimmunol.177.5.3201
	HIV-1-infected macrophages	human	HIV-1 Tat protein	PGE_2_ ↑	↑	HIV-1-infected macrophages enhance *Leishmania* replication	^64^	10.1086/506618
	coculture: monocyte-derived dendritic cells and neutrophils	human	fibronectin, DC-SIGN neutralizing antibody	PGE_2_ ↑	↓	neutrophil–DC interaction via DC-SIGN increases PGE_2_ and eliminates parasites	^54^	10.3389/fimmu.2021.750648
	peritoneal macrophages	BALB/c, C57BL/6 and C57BL/6 P2X7^–/–^ mice	LTB_4_, LTC_4_, LTD4, ATP	LTC_4_, LTD_4_, and LTE_4_ (Cys-LTs)	↓	Cys-LTs reduced *L. amazonensis* infection, an effect dependent on the P2X7 receptor	^58^	10.3389/fcimb.2023.1192800
	neutrophil and macrophage	human	indomenthacin	-	↑	effect apoptotic neutrophils enhances parasite load via COX-2 activation	^26^	10.1189/jlb.0108018
*Leishmania major*	T cells	BALB/c, C57BL/6 and CBA mice	-	PGE_2_ ↑	-	susceptible BALB/c T cells produce more PGE_2_, IL-1, and TGF-β	^88^	10.1128/iai.65.7.2837-2845.1997
	B-1CDP	BALB/c	COX inhibitors	PGE_2_ ↑	↑	PGE_2_-induced IL-10 drives B-1CDP susceptibility to infection	^89^	10.1371/journal.pone.0124888
	neutrophils and HT-29	human and human epithelial cell line	ionomycin, LPS and fMLP	LTB_4_ ↑, LXA_4_ ↓	-	infected neutrophils exhibit higher LTB_4_ and lower LXA_4_ levels	^25^	10.1155/2017/2014583
*Leishmania mexicana*	spleen cells	BALB/c e C57BL/6 mice	INDO,SLA	PGE_2_ ↓	↓	INDO enhances Th1 response, inhibits PGE_2_ and kills the parasites	^87^	10.1046/j.1365-3024.2001.00421.x
	J774 macrophage	murine cell line	PPAR agonist, cPLA_2_ inhibitor	↑: PGE_2_ and PGF_2 α_ ↓: 6k-PGF_1_, PGE_1α_ and PGF_1_	↓	PPAR_Y_ inhibits cPLA2-COX-2, increases ROS, PGE_2_ and PGF_2α_ against *L. mexicana*	^67^	10.1155/2013/215283
*Leishmania braziliensis*	macrophage	human with CL	PGE_2_, COX inhibitor	PGE_2_ ↓	↓	PGE_2_ inhibition controls inflammation and *L. braziliensis* infection	^66^	10.1080/22221751.2023.2261565
	macrophage	human	LTB_4_, FLAP inhibitor, BLT1 antagonist	LTB_4_ ↑	↓	*L. braziliensis* induces LTB_4_, which rapidly controls infection	^65^	10.1016/j.micinf.2014.08.015
	macrophage	human with CL + DM or with CL only	glycose	PGE_2_ ↑ and LTB_4_ ↓	↑	CL+DM macrophages favor infection by increasing PGE_2_ over LTB_4_	^68^	10.1080/22221751.2020.1773744

a Acronyms: AA—arachidonic acid; ATP—adenosine triphosphate; B-1CDP—B-1 cell development progentors; BLT1—leukotriene B1 receptor; BMDM—bone marrow-derived macrophages; CL—cutaneous leishmaniasis; Con A—concanavalin A; COX-2—cyclooxygenase-2; cPLA_2_—cytosolic phospholipase A_2_; DC—dendritic cells; DC-SIGN—dendritic cell-specific intercellular adhesion molecule-3-grabbing nonintegrin; DHA—docosahexaenoic acid; DK-PGD_2_—13,14-dihydro-15-keto prostaglandin D_2_; DM—diabetes mellitus; DP2—prostaglandin D_2_ receptor 2; dPGJ_2_—deoxy-δ. Prostaglandin J_2_; EET—Epoxygenated Derivatives of Arachidonic Acid; EpETrE—Epoxyeicosatrienoic acid; FLAP—5-lipoxygenase-activating protein; fMLP—N-formylmethionyl-leucyl-phenylalanine; GA—glycyrrhizic acid; HDoHE—hydroxydocosahexaenoic acid; HETE—hydroxyeicosatetraenoic acid; HIV-1—human immunodeficiency virus type 1; HT-29—human colorectal cancer cell line; IFN-_Y_—interferon γ; IL-1beta—interleukin-1 β; IL-ip—interleukin-1 β; IL-2—interleukin-2; INDO—indomethacin; LA—linoleic acid; LA—linoleic acid; LO—lipoxygenase; LPS—lipophosphoglycan; LPS—lipopolysaccharide; LTA_4_H—leukotriene A_4_ hydrolase; LTB_4_—leukotriene B4; LXA_4_—lipoxin A_4_; NLRP3—nod-like receptor pyrin domain-containing 3; NO—nitric oxide; ODC—ornithine decarboxylase; ODC—ornithine decarboxylase; P2X7—P2X7 receptor; PDx—protectin D; PGD_2_—prostaglandin D_2_; PGE_2_—prostaglandin E2; PGF2a—prostaglandin F_2__α_; PKC-alfa—protein kinase C-α; PLA_2_—phospholipase A_2_; PPAR*y*—peroxisome proliferator-activated receptor γ; ROS—reactive oxygen species; RvD_2_—resolvin D_2_; SGS—salivary gland sonicate; siRNA—small interfering RNA; SLA—soluble leishmania antigen; TLR—toll-like receptor.

### Lipid Mediator Roles in Experimental Models of *Leishmania* Infection

The use of in vivo experimental models allows the observation of complex processes involving host–parasite interactions, pathogenesis, drug efficacy, vaccine development.^90,91^ In the context of *Leishmania* infections, mice strains, such as BALB/c and C57BL/6 (*Mus musculus*), as well as golden hamsters (*Mesocricetus auratus*), are widely used in studies of CL and VL. These models exhibit distinct immunological profiles that determine their susceptibility or resistance to infection.^92^ For example, BALB/c mice are susceptible to *L. major*, whereas C57BL/6 mice are resistant.^92^ In VL, golden hamsters closely mimic active disease in dogs and humans; however, BALB/c and C57BL/6 mice are also susceptible and are extensively used in vaccine research.^93^ This section addresses the main aspects of animal species and strains employed as experimentals models in leishmaniasis research, with a focus on their utility for in vivo analyses of lipid precursors and mediators that modulate host–parasite interactions.

#### *Mus musculus* Inbred Strain BALB/c

In vivo studies in BALB/c mice demonstrate that interventions modulating inflammatory mediators significantly influence the course of cutaneous *Leishmania* infections. PLA_2_-treated *L. amazonensis* promastigotes increased lesion size and induced necrotic areas rich in polymorphonuclear, mononuclear, and amastigote cells.^62^ Consistently, pharmacological or genetic blockade of endogenous leukotriene synthesis enhanced footpad swelling in susceptible mice.^60^ Additionally, in *L. mexicana* infection, lesions began to develop from week 8, associated with a Th2 response characterized by high IL-4 and IL-10 production, while INDO treatment delayed lesion progression.^87^ These findings indicate that modulation of inflammatory mediators, such as LTs and PGs, plays a central role in the cutaneous lesion progression in BALB/c mice.

In the context of VL, nutritional and pharmacological factors significantly modulate the infection progression. Malnutrition in mice infected with *L. donovani* increased visceralization, reduced NO production, and elevated PGE_2_, correlating with higher parasite burden.^75^ In contrast, high-fat-high-cholesterol diets in mice were infected with *L. infantum* reduced hepatic parasite burden, although they increased inflammation and the expression of lipid mediator-related genes such as Alox5, Alox12, and Alox15.^94^ Pharmacological interventions also influenced infection outcomes: GA administration reduced myeloid-derived suppressor cells (MDSCs), COX-2, and PGE_2_ levels,^95^ while 15d-PGJ_2_ exhibited leishmanicidal activity in the spleen and liver, allowing for lower doses of miltefosine and amphotericin B.^80^ Additionally, blocking the PGE_2_ pathway with NS-398 or AH6809 decreased parasite burden and spleen weight, indicating that overproduction of this mediator favors parasite survival by attenuating inflammatory and Th17 responses.^69^

#### *Mus musculus* Inbred Strain C57BL/6

In experimental models of CL, the characteristic resistance of C57BL/6 mice has also been associated with *L. mexicana* infection. After 8 weeks, the animals displayed stable footpad lesion size and a Th1-type immune response, characterized by high IL-2 and IFN-γ production, without an increase in PGE_2_.^87^ More recent findings have expanded the understanding of these mechanisms, showing that *L. mexicana* infection also induces distinct metabolic signatures in auricular tissue, including increased AA metabolites and their precursors, with potential anti-inflammatory and antinociceptive effects.^96^ Similarly, in *L. amazonensis* infections, lipid mediators played a central role in infection control. Cys-LTs reduced lesion size and parasite burden in the footpads, highlighting them as potential therapeutic targets.^58^ Furthermore, local administration of LTB_4_ restored resistance in P2X7^–/–^ mice, as evidenced by decreased lesion size and parasite load.^59^

Metabolic alterations were also observed in the experimental models of VL. During *L. donovani* infection, mass spectrometry imaging revealed heterogeneous lipid distribution in the liver of infected mice, with 15 lipids unique to infected livers, highlighting the dynamic role of lipids in host response.^97^ In *L. infantum* infection, C-type lectin receptors (CLRs), such as Dectin-1 and MR, proved crucial for controlling parasitemia, as mice deficient in these receptors exhibited higher parasite loads in blood and spleen. These findings emphasize the importance of CLRs in VL and suggest their potential as therapeutic targets.^79^

#### *Mesocricetus auratus*—Golden Hamster

In hamsters, *Leishmania* infection induces marked metabolic and therapeutic responses. During *L. donovani* infection, tissue-specific alterations were observed: COX-2 and PGE_2_ synthases (cPGES/mPGES) increased mainly in the lymph node, liver, and spleen (with COX-2 peaking in the spleen at 60 dpi), while mPGES decreased in the bone marrow; 5-LO was elevated in the liver and lymph node but reduced in the spleen and bone marrow.^83^ In parallel, LA accumulated in the spleen and liver, and AA increased in the spleen, liver, and lymph node, while both remained unchanged or undetectable in the bone marrow.^83^ Similarly, *L. infantum* infection remodels hepatic phospholipids and enhances membrane and AA metabolism through the upregulation of PLA_2_, COX-2, and Alox5.^98^ Moreover, the imbalance between HETEs and PGs, characterized by increased AA, 5-HETE, 8-HETE, 11-HETE, 12-HETE, 15-HETE, LTB_4_ and reduced PGE_2_, 2-keto-PGE_2_, and PGD_2_ in the spleen, correlated with splenomegaly and parasite burden, indicating that *L. infantum* exploits this shift to sustain inflammation and disease progression.^99^ Regarding treatment, administration of 15d-PGJ_2_ (4 mg/kg/day) to *L. donovani*-infected hamsters reduced splenic and hepatic parasite burdens, reaching maximum inhibition of 89% and 83% at day 7 post-treatment; when combined with subcurative doses of Miltefosine or Amphotericin B, inhibition increased to 92–95%, demonstrating that 15d-PGJ_2_ enhances antileishmanial efficacy and allows the dose reduction of conventional drugs in experimental visceral leishmaniasis.^80^

### Lipid Mediators and Leishmaniasis Clinical Presentations

Patients with different clinical forms of leishmaniasis exhibit distinct lipid mediator profiles, reflecting the heterogeneity of the immune response and providing insights into biomarker discovery, therapeutic failure, and treatment strategies. In this context of the CL, specifically when compared with healthy individuals, patients with LCL form showed increased plasma PGE_2_ along with reduced PGD_2_, PGF_2_α, LTB_4_, and RvD_1_; patients with DCL displayed elevated plasma PGE_2_ levels;^61^ and those with MCL exhibited a decrease only in PGF_2α_.^100^ Consistently, the PGE_2_ pathway is strongly upregulated in active CL lesions, where high PTGS2/COX-2 expression and elevated PGE_2_ production by resident cells correlate with increased parasite burden, greater number of lesions, disease severity, and treatment failure, highlighting the detrimental role of PGE_2_ signaling in disease progression and poor clinical outcomes.^66^ In addition, a biosignature enriched in lipid mediators was shown to reliably predict treatment outcomes in CL: 60 days after therapy, cured patients had significantly lower plasma levels of LTB_4_, 5-oxo-HETE, 12-oxo-HETE, 12-HETE, 11-HETE, and 15-HETE compared to those with treatment failure, supporting their potential as targets for host-directed therapies.^101^

Other conditions, such as diabetes mellitus (DM), can exacerbate the systemic inflammatory profile in the CL. In this context, individuals with CL + DM showed elevated plasma levels of LTB_4_ compared to those with CL alone. Moreover, glycemic levels correlated with both serum LTB_4_ levels and lesion healing times. There was also a reduction in ALOX5 expression in the lesions of patients with CL + DM compared with those with CL. Together, these findings suggest that LTB_4_ may represent a potential therapeutic target to mitigate the impact of leishmaniasis in individuals with diabetes.^68^

Comparative analyses of the clinical forms of CL revealed distinct inflammatory and lipid mediator profiles. In situ analyses of mucosal and skin biopsies showed that patients with LCL exhibited higher expression of prostaglandin pathway genes (PGES, PTGER3, PGDS, PTGFR, PTGS1), whereas those with MCL had increased expression of LXA_4_R, highlighting divergent lipid mediator signatures that may serve as biomarkers of active disease and clinical outcome.^100^ Additionally, plasma RvD_1_ levels distinguished patients with DCL from those with LCL, being significantly elevated in DCL and positively correlated to the number of lesions. These results suggest that RvD_1_ contributes to the attenuated inflammatory response observed in DCL and may represent a potential target for host-directed therapy.^102^

Lipid mediator dynamics also play a pivotal role in VL. Active VL was associated with elevated serum PGF_2α_, LTB_4_, and RvD_1_, which progressively declined after therapy, underscoring treatment-driven modulation of the inflammatory environment.^103^ Furthermore, LA levels were significantly reduced in the serum of patients with VL compared to healthy individuals, indicating additional alterations in the lipid mediator profile during the disease.^83^ These findings underscore that lipid mediator imbalance reflects both the pathogenesis and the treatment response in HVL. In CVL, increasing disease severity was associated with a progressive decrease in LTB_4_ and PGE_2_ levels in the serum of affected dogs. Moreover, the combination of LTB_4_, PGE_2_, and CXCL1 accurately distinguished dogs with different severity levels, suggesting that clinical severity in CVL reflects a specific inflammatory profile, characterized by alterations in circulating eicosanoids and chemokines.^104^ Collectively, these findings demonstrate that lipid mediators modulate the immunopathological spectrum of leishmaniasis, influencing clinical outcomes, comorbidities, and treatment responses. Their distinct profiles highlight their potential as biomarkers and as targets for host-directed therapeutic interventions. The lipid mediators linked to the various clinical manifestations of leishmaniasis are illustrated in Figure 1.

## Conclusion

Research on lipid mediators in leishmaniasis has revealed their central role in regulating host–parasite interactions, influencing both the resistance and susceptibility to infection. Eicosanoids such as PGE_2_ and LTB_4_ emerge as key determinants of the disease outcome, acting in a balance between parasite persistence and elimination. Experimental models and clinical studies demonstrate that lipid mediator profiles vary according to the Leishmania species, host cell type, and clinical form of the disease, reinforcing the complexity of the immunometabolic regulation in this context.

Despite significant advances, important gaps in knowledge remain. Some lipid mediators, including PGF_2α_, LXA_4_, thromboxanes, and non-AA-derived molecules, have been little studied in leishmaniasis and may possess as yet unexplored immunomodulatory potential. Furthermore, the role of HETEs in modulating inflammation and treatment resistance requires further investigation. However, the growing application of metabolomic and lipidomic approaches, combined with mass spectrometry quantification, has expanded our understanding of lipid mediator networks and revealed new candidates for biomarker discovery and host-directed therapies. Therefore, the integration of experimental, clinical, and systems biology approaches is essential to elucidate how lipid mediators orchestrate immune responses in leishmaniasis. These advances may provide the basis for the development of innovative therapeutic strategies aimed at modulating host immunity and improving disease control.

## References

[1] Taslimi Y., Zahedifard F., Rafati S. (2018). Leishmaniasis and Various Immunotherapeutic
Approaches. Parasitology.

[2] Mann S., Frasca K., Scherrer S., Henao-Martínez A. F., Newman S., Ramanan P., Suarez J. A. (2021). A Review of Leishmaniasis:
Current Knowledge and Future Directions. Curr.
Trop. Med. Rep..

[3] Burza S., Croft S. L., Boelaert M. (2018). Leishmaniasis. Lancet.

[4] Cosma C., Maia C., Khan N., Infantino M., Del Riccio M. (2024). Leishmaniasis in Humans and Animals:
A One Health Approach
for Surveillance, Prevention and Control in a Changing World. Trop. Med. Infect. Dis..

[5] López-Muñoz R. A., Molina-Berríos A., Campos-Estrada C., Abarca-Sanhueza P., Urrutia-Llancaqueo L., Peña-Espinoza M., Maya J. D. (2018). Inflammatory and Pro-Resolving Lipids
in Trypanosomatid
Infections: A Key to Understanding Parasite Control. Front. Microbiol..

[6] Melo R. C. N., Weller P. F. (2016). Lipid Droplets in Leukocytes: Organelles
Linked to
Inflammatory Responses. Exp. Cell Res..

[7] Samuelsson B. (2012). Role of Basic
Science in the Development of New Medicines: Examples from the Eicosanoid
Field. J. Biol. Chem..

[8] Serhan C. N., Chiang N., Dalli J., Levy B. D. (2015). Lipid Mediators
in the Resolution of Inflammation. Cold Spring
Harbor Perspect. Biol..

[9] Stables M. J., Gilroy D. W. (2011). Old and New Generation Lipid Mediators
in Acute Inflammation
and Resolution. Prog. Lipid Res..

[10] Dyall S. C., Balas L., Bazan N. G., Brenna J. T., Chiang N., da Costa Souza F., Dalli J., Durand T., Galano J. M., Lein P. J., Serhan C. N., Taha A. Y. (2022). Polyunsaturated
Fatty Acids and Fatty Acid-Derived Lipid Mediators: Recent Advances
in the Understanding of Their Biosynthesis, Structures, and Functions. Prog. Lipid Res..

[11] Chaves M. M., Canetti C., Coutinho-Silva R. (2016). Crosstalk
between Purinergic Receptors
and Lipid Mediators in Leishmaniasis. Parasites
Vectors.

[12] Guimaraes-Costa A. B., Shannon J. P., Waclawiak I., Oliveira J., Meneses C., de Castro W., Wen X., Brzostowski J., Serafim T. D., Andersen J. F., Hickman H. D., Kamhawi S., Valenzuela J. G., Oliveira F. (2021). A Sand Fly Salivary
Protein Acts
as a Neutrophil Chemoattractant. Nat. Commun..

[13] Peters N. C., Egen J. G., Secundino N., Debrabant A., Kimblin N., Kamhawi S., Lawyer P., Fay M. P., Germain R. N., Sacks D. (2008). In Vivo Imaging Reveals an Essential
Role for Neutrophils in Leishmaniasis Transmitted by Sand Flies. Science.

[14] Goncalves R., Zhang X., Cohen H., Debrabant A., Mosser D. M. (2011). Platelet Activation Attracts a Subpopulation
of Effector
Monocytes to Sites of Leishmania Major Infection. J. Exp. Med..

[15] Ribeiro-Gomes F. L., Sacks D. (2012). The Influence of Early
Neutrophil-Leishmania Interactions on the
Host Immune Response to Infection. Front. Cell.
Infect. Microbiol..

[16] Thalhofer C. J., Chen Y., Sudan B., Love-Homan L., Wilson M. E. (2011). Leukocytes Infiltrate the Skin and Draining Lymph Nodes
in Response to the Protozoan Leishmania Infantum Chagasi. Infect. Immun..

[17] Wei F., Gong W., Wang J., Yang Y., Liu J., Wang Y., Cao J. (2019). Role of the
Lipoxin A4 Receptor in
the Development of Neutrophil Extracellular Traps in Leishmania Infantum
Infection. Parasites Vectors.

[18] Rossi M., Fasel N. (2018). How to Master the Host
Immune System? Leishmania Parasites Have the
Solutions!. Int. Immunol..

[19] Costa-da-silva A. C., Nascimento D. D. O., Ferreira J. R. M., Guimar K., Freire-de-lima L., Morrot A., Decote-ricardo D., Filardy A. A., Freire-de-lima C. G. (2022). Immune
Responses in Leishmaniasis : An Overview. Trop. Med. Infect. Dis..

[20] Teixeira C. R., da S Santos C., Prates D. B., dos Santos R. T., Araújo-Santos T., de Souza-Neto S. M., Borges V. M., Barral-Netto M., Brodskyn C. I. (2018). Lutzomyia Longipalpis Saliva Drives Interleukin-17-Induced
Neutrophil Recruitment Favoring Leishmania Infantum Infection. Front. Microbiol..

[21] Prates D. B., Araújo-Santos T., Luz N. F., Andrade B. B., França-Costa J., Afonso L., Clarêncio J., Miranda J. C., Bozza P. T., DosReis G. A., Brodskyn C., Barral-Netto M., de Matos Borges V., Barral A. (2011). Lutzomyia Longipalpis Saliva Drives
Apoptosis and Enhances Parasite Burden in Neutrophils. J. Leukocyte Biol..

[22] Araújo-Santos T., Prates D. B., França-Costa J., Luz N. F., Andrade B. B., Miranda J. C., Brodskyn C. I., Barral A., Bozza P. T., Borges V. M. (2014). Prostaglandin
E2/Leukotriene B4 Balance Induced by
Lutzomyia Longipalpis Saliva Favors Leishmania Infantum Infection. Parasites Vectors.

[23] Tavares N. M., Araújo-Santos T., Afonso L., Nogueira P. M., Lopes U. G., Soares R. P., Bozza P. T., Bandeira-Melo C., Borges V. M., Brodskyn C. (2014). Understanding
the Mechanisms Controlling
Leishmania Amazonensis Infection in Vitro: The Role of LTB4derived
from Human Neutrophils. J. Infect. Dis..

[24] Tavares N., Afonso L., Suarez M., Ampuero M., Prates D. B., Araújo-Santos T., Barral-Netto M., DosReis G. A., Borges V. M., Brodskyn C. (2016). Degranulating Neutrophils Promote Leukotriene B4 Production
by Infected Macrophages To Kill Leishmania Amazonensis Parasites. J. Immunol..

[25] Plagge M., Laskay T. (2017). Early Production of
the Neutrophil-Derived Lipid Mediators
LTB4 and LXA4 Is Modulated by Intracellular Infection with Leishmania
Major. BioMed Res. Int..

[26] Afonso L., Borges V. M., Cruz H., Ribeiro-Gomes F. L., DosReis G. A., Dutra A. N., Clarêncio J., de Oliveira C. I., Barral A., Barral-Netto M., Brodskyn C. I. (2008). Interactions with Apoptotic but Not with Necrotic Neutrophils
Increase Parasite Burden in Human Macrophages Infected with Leishmania
Amazonensis. J. Leukocyte Biol..

[27] Lauvau G.., Loke P., Hohl T. M. (2015). Monocyte-Mediated
Defense against
Bacteria, Fungi, and Parasites. Semin. Immunol..

[28] Shi C., Pamer E. G. (2011). Monocyte Recruitment
during Infection and Inflammation. Nat. Rev.
Immunol..

[29] Glennie N. D., Volk S. W., Scott P. (2017). Skin-Resident CD4+T Cells Protect
against Leishmania Major by Recruiting and Activating Inflammatory
Monocytes. PLoS Pathog..

[30] Figueira C. P., Carvalhal D. G. F., Almeida R. A., D’El-Rei Hermida M., Touchard D., Robert P., Pierres A., Bongrand P., Dos-Santos W. L. C. (2015). Leishmania
Infection Modulates Beta-1 Integrin Activation
and Alters the Kinetics of Monocyte Spreading over Fibronectin. Sci. Rep..

[31] de
Freitas R. C., Lonien S. C. H., Malvezi A. D., Silveira G. F., Wowk P. F., da Silva R. V., Yamauchi L. M., Yamada-Ogatta S. F., Rizzo L. V., Bordignon J., Pinge-Filho P. (2017). Trypanosoma
Cruzi: Inhibition of Infection of Human Monocytes by Aspirin. Exp. Parasitol..

[32] Rothenberg M. E., Hogan S. P. (2006). The Eosinophil. Annu. Rev. Immunol..

[33] Knuplez E., Sturm E. M., Marsche G. (2021). Emerging Role
of Phospholipase-Derived
Cleavage Products in Regulating Eosinophil Activity: Focus on Lysophospholipids,
Polyunsaturated Fatty Acids and Eicosanoids. Int. J. Mol. Sci..

[34] Lee J. J., Jacobsen E. A., McGarry M. P., Schleimer R. P., Lee N. A. (2010). Eosinophils in Health
and Disease: The LIAR Hypothesis. Clin. Exp.
Allergy.

[35] Acharya K. R., Ackerman S. J. (2014). Eosinophil Granule
Proteins: Form and Function. J. Biol. Chem..

[36] Blom M., Tool A. T. J., Wever P. C., Wolbink G. J., Brouwer M. C., Calafat J., Egesten A., Knol E. F., Hack C. E., Roos D., Verhoeven A. J. (1998). Human Eosinophils Express, Relative
to Other Circulating Leukocytes, Large Amounts of Secretory 14-KD
Phospholipase A2. Blood.

[37] Bowton D. L., Seeds M. C., Fasano M. B., Goldsmith B., Bass D. A. (1997). Phospholipase A2 and Arachidonate
Increase in Bronchoalveolar
Lavage Fluid after Inhaled Antigen Challenge in Asthmatics. Am. J. Respir. Crit. Care Med..

[38] Sokolowska M., Stefanska J., Wodz-Naskiewicz K., Cieslak M., Pawliczak R. (2010). Cytosolic
Phospholipase A2 Group IVA Is Overexpressed in Patients with Persistent
Asthma and Regulated by the Promoter Microsatellites. J. Allergy Clin. Immunol..

[39] De
Oliveira Cardoso F., Da Silva Freitas De Souza C., Mendes V. G., Abreu-Silva A. L., Da Costa S. C. G., Da Silva Calabresa K. (2010). Immunopathological
Studies of Leishmania Amazonensis Infection in Resistant and in Susceptible
Mice. J. Infect. Dis..

[40] Grimaldi G., Soares M. J., Moriearty P. L. (1984). Tissue
Eosinophilia and Leishmania Mexicana Mexicana Eosinophil Interactions
in Murine Cutaneous Leishmaniasis. Parasite
Immunol..

[41] Pearson R. D., Uydess I. L., Chapman S. W., Steigbigel R. T. (1987). Interaction
of Human Eosinophils with Leishmania Donovani. Ann. Trop. Med. Parasitol..

[42] Saito S., Hamada A., Watanabe N., Obata T., Katakura K., Ohtomo H. (1996). Eosinophil Chemotactic
Activity in Leishmania Amazonensis
Promastigotes. Parasitol. Res..

[43] Menezes-Souza D., Guerra-Sá R., Carneiro C. M., Vitoriano-Souza J., Giunchetti R. C., Teixeira-Carvalho A., Silveira-Lemos D., Oliveira G. C., Corrêa-Oliveira R., Reis A. B. (2012). Higher
Expression of CCL2, CCL4, CCL5, CCL21, and CXCL8 Chemokines in the
Skin Associated with Parasite Density in Canine Visceral Leishmaniasis. PLoS Neglected Trop. Dis..

[44] Monteiro M. C., Lima H. C., Souza A. A. A., Titus R. G., Romão P. R. T., de Queiroz Cunha F. (2007). Effect of Lutzomyia Longipalpis Salivary
Gland Extracts on Leukocyte Migration Induced by Leishmania Major. Am. J. Trop. Med. Hyg..

[45] da
Silva Marques P., da Fonseca-Martins A.
M., Carneiro M. P. D., Amorim N. R. T., de Pão C. R. R., Canetti C., Diaz B. L., de Matos Guedes H. L., Bandeira-Melo C. (2021). Eosinophils Increase Macrophage Ability
to Control Intracellular Leishmania Amazonensis Infection via PGD2
Paracrine Activity in Vitro. Cell. Immunol..

[46] Brandonisio O., Spinelli R., Pepe M. (2004). Dendritic
Cells in Leishmania Infection. Microbes Infect..

[47] Tibúrcio R., Nunes S., Nunes I., Ampuero M. R., Silva I. B., Lima R., Tavares N. M., Brodskyn C. (2019). Molecular Aspects of
Dendritic Cell Activation in Leishmaniasis: An Immunobiological View. Front. Immunol..

[48] Gualde N., Harizi H. (2004). Prostanoids and Their Receptors That
Modulate Dendritic
Cell-Mediated Immunity. Immunol. Cell Biol..

[49] Ohba M., Saeki K., Koga T., Okuno T., Kobayashi Y., Yokomizo T. (2018). Profiling of Bioactive Lipids in Different Dendritic
Cell Subsets Using an Improved Multiplex Quantitative LC-MS/MS Method. Biochem. Biophys. Res. Commun..

[50] Duffney P. F., Falsetta M. L., Rackow A. R., Thatcher T. H., Phipps R. P., Sime P. J. (2018). Key Roles for Lipid
Mediators in the Adaptive Immune
Response. J. Clin. Invest..

[51] Figueiredo A. B., Serafim T. D., Marques-da-Silva E. A., Meyer-Fernandes J. R., Afonso L. C. C. (2012). Leishmania Amazonensis Impairs DC
Function by Inhibiting
CD40 Expression via A 2B Adenosine Receptor Activation. Eur. J. Immunol..

[52] Steigerwald M., Moll H. (2005). Leishmania Major Modulates
Chemokine and Chemokine Receptor Expression
by Dendritic Cells and Affects Their Migratory Capacity. Infect. Immun..

[53] Hermida M. D. R., Doria P. G., Taguchi A. M. P., Mengel J. O., dos-Santos W. L. C. (2014). Leishmania
Amazonensis Infection Impairs Dendritic Cell Migration from the Inflammatory
Site to the Draining Lymph Node. BMC Infect.
Dis..

[54] Tiburcio R., Melo L. D., Nunes S., Barbosa A. L. A., de
Oliveira E. C., Suarez M., Borges V. M., Tavares N., Brodskyn C. I. (2021). DC-SIGN Mediates the Interaction Between Neutrophils
and Leishmania Amazonensis-Infected Dendritic Cells to Promote DC
Maturation and Parasite Elimination. Front.
Immunol..

[55] Wynn T. A., Chawla A., Pollard J. W. (2013). Macrophage Biology in Development,
Homeostasis and Disease. Nature.

[56] Liu D., Uzonna J. E. (2012). The Early
Interaction of Leishmania with Macrophages
and Dendritic Cells and Its Influence on the Host Immune Response. Front. Cell. Infect. Microbiol..

[57] Chaves M. M., Marques-da-Silva C., Monteiro A. P. T., Canetti C., Coutinho-Silva R. (2014). Leukotriene
B4Modulates P2 × 7 Receptor–Mediated Leishmania Amazonensis
Elimination in Murine Macrophages. J. Immunol..

[58] Noronha L. P. T., Martins M. D. A., Castro-Junior A. B., Thorstenberg M. L., Costa-Soares L., Rangel T. P., Carvalho-Gondim F., Rossi-Bergmann B., Savio L. E. B., de Azevedo Canetti C., Coutinho-Silva R. (2023). Cysteinyl-Leukotrienes Promote Cutaneous Leishmaniasis
Control. Front. Cell. Infect. Microbiol..

[59] Chaves M. M., Sinflorio D. A., Thorstenberg M. L., Martins M. D. A., Moreira-Souza A. C. A., Rangel T. P., Silva C. L. M., Bellio M., Canetti C., Coutinho-Silva R. (2019). Non-Canonical NLRP3 Inflammasome Activation and Il-1β
Signaling Are Necessary to L. Amazonensis Control Mediated by P2 ×
7 Receptor and Leukotriene B4. PLoS Pathog..

[60] Serezani C. H., Perrela J. H., Russo M., Peters-Golden M., Jancar S. (2006). Leukotrienes Are Essential for the
Control of Leishmania
Amazonensis Infection and Contribute to Strain Variation in Susceptibility. J. Immunol..

[61] França-Costa J., Van Weyenbergh J., Boaventura V. S., Luz N. F., Malta-Santos H., Oliveira M. C. S., De Campos D. C. S., Saldanha A. C., Dos-Santos W. L. C., Bozza P. T., Barral-Netto M., Barral A., Costa J. M., Borges V. M. (2015). Arginase I, Polyamine, and Prostaglandin E2 Pathways
Suppress the Inflammatory Response and Contribute to Diffuse Cutaneous
Leishmaniasis. J. Infect. Dis..

[62] Passero L. F. D., Laurenti M. D., Tomokane T. Y., Corbett C. E. P., Toyama M. H. (2008). The Effect
of Phospholipase A2 from Crotalus Durissus Collilineatus on Leishmania
(Leishmania) Amazonensis Infection. Parasitol.
Res..

[63] Pinheiro R. O., Nunes M. P., Pinheiro C. S., D’Avila H., Bozza P. T., Takiya C. M., Côrte-Real S., Freire-de-Lima C. G., DosReis G. A. (2009). Induction of Autophagy Correlates
with Increased Parasite Load of Leishmania Amazonensis in BALB/c but
Not C57BL/6 Macrophages. Microbes Infect..

[64] Barreto-De-Souza V., Pacheco G. J., Silva A. R., Castro-Faria-Neto H. C., Bozza P. T., Saraiva E. M., Bou-Habib D. C. (2006). Increased
Leishmania Replication in HIV-1-Infected Macrophages Is Mediated by
Tat Protein through Cyclooxygenase-2 Expression and Prostaglandin
E 2 Synthesis. J. Infect. Dis..

[65] Morato C.
I., da Silva I. A., Borges A. F., Dorta M. L., Oliveira M. A. P., Jancar S., Serezani C. H., Ribeiro-Dias F. (2014). Essential
Role of Leukotriene B4 on Leishmania (Viannia) Braziliensis Killing
by Human Macrophages. Microbes Infect..

[66] Nascimento M. T., Viana D. L., Peixoto F. C., Arruda S. M., Carvalho E. M., Carvalho L. P. (2023). Prostaglandin E2 Contributes to L. Braziliensis Survival
and Therapeutic Failure in Cutaneous Leishmaniasis. Emerging Microbes Infect..

[67] Díaz-Gandarilla J. A., Osorio-Trujillo C., Hernández-Ramírez V. I., Talamás-Rohana P. (2013). PPAR Activation
Induces M1Macrophage
Polarization via CPLACOX-2 Inhibition, Activating Ros Production against
Leishmania Mexicana. Biomed Res. Int..

[68] Bonyek-Silva I., Nunes S., Santos R. L., Lima F. R., Lago A., Silva J., Carvalho L. P., Arruda S. M., Serezani H. C., Carvalho E. M., Brodskyn C. I., Tavares N. M. (2020). Unbalanced Production
of LTB4/PGE2 Driven by Diabetes Increases Susceptibility to Cutaneous
Leishmaniasis. Emerging Microbes Infect..

[69] Saha A., Biswas A., Srivastav S., Mukherjee M., Das P. K., Ukil A. (2014). Prostaglandin E2 Negatively Regulates
the Production of Inflammatory Cytokines/Chemokines and IL-17 in Visceral
Leishmaniasis. J. Immunol..

[70] Giroux M., Descoteaux A. (2000). Cyclooxygenase-2
Expression in Macrophages: Modulation
by Protein Kinase C-α. J. Immunol..

[71] Blot C., Lavernhe M., Lugo-Villarino G., Coulson K., Salon M., Tertrais M., Planès R., Santoni K., Authier H., Jacquemin G., Rahabi M., Parny M., Letron I. R., Meunier E., Lefèvre L., Coste A. (2024). Leishmania Infantum
Exploits the Anti-Ferroptosis Effects of Nrf2 to Escape Cell Death
in Macrophages. Cell Rep.

[72] Reiner N. E., Malemud C. J. (1985). Arachidonic Acid
Metabolism by Murine Peritoneal Macrophages
Infected with Leishmania Donovani: In Vitro Evidence for Parasite-Induced
Alterations in Cyclooxygenase and Lipoxygenase Pathways. J. Immunol..

[73] Reiner N. E., Ng W., McMaster W. R. (1987). Parasite-Accessory
Cell Interactions in Murine Leishmaniasis.
II. Leishmania Donovani Suppresses Macrophage Expression of Class
I and Class II Major Histocompatibility Complex Gene Products. J. Immunol..

[74] Paloque L., Perez-Berezo T., Abot A., Dalloux-Chioccioli J., Bourgeade-Delmas S., Le Faouder P., Pujo J., Teste M. A., François J. M., Schebb N. H., Mainka M., Rolland C., Blanpied C., Dietrich G., Bertrand-Michel J., Deraison C., Valentin A., Cenac N. (2019). Polyunsaturated Fatty
Acid Metabolites: Biosynthesis in Leishmania and Role in Parasite/Host
Interaction. J. Lipid Res..

[75] Anstead G. M., Zhang Q., Melby P. C. (2009). Malnutrition
Promotes Prostaglandin
over Leukotriene Production and Dysregulates Eicosanoid-Cytokine Crosstalk
in Activated Resident Macrophages. Prostaglandins,
Leukotrienes Essent. Fatty Acids.

[76] Araújo-Santos T., Rodríguez N. E., Moura-Pontes S., Dixt U. G., Abánades D. R., Bozza P. T., Wilson M. E., Borges V. M. (2014). Role of Prostaglandin
F < inf > 2α</Inf> Production in Lipid Bodies from
Leishmania
Infantum Chagasi: Insights on Virulence. J.
Infect. Dis..

[77] Bhattacharjee A., Majumder S., Das S., Ghosh S., Biswas S., Majumdar S. (2016). Leishmania Donovani-Induced Prostaglandin E2 Generation
Is Critically Dependent on Host Toll-Like Receptor 2–Cytosolic
Phospholipase A2 Signaling. Infect. Immun..

[78] Lima J. B., Araújo-Santos T., Lázaro-Souza M., Carneiro A. B., Ibraim I. C., Jesus-Santos F. H., Luz N. F., Pontes S. D. M., Entringer P. F., Descoteaux A., Bozza P. T., Soares R. P., Borges V. M. (2017). Leishmania
Infantum Lipophosphoglycan Induced-Prostaglandin E2production in Association
with PPAR-γ Expression via Activation of Toll like Receptors-1
and 2. Sci. Rep..

[79] Lefèvre L., Lugo-Villarino G., Meunier E., Valentin A., Olagnier D., Authier H., Duval C., Dardenne C., Bernad J., Lemesre J. L., Auwerx J., Neyrolles O., Pipy B., Coste A. (2013). The C-Type
Lectin Receptors Dectin-1,
MR, and SIGNR3 Contribute Both Positively and Negatively to the Macrophage
Response to Leishmania Infantum. Immunity.

[80] Vishwakarma P., Parmar N., Yadav P. K., Chandrakar P., Kar S. (2016). 15d-Prostaglandin J2 Induced Reactive Oxygen Species-Mediated Apoptosis
during Experimental Visceral Leishmaniasis. J. Mol. Med..

[81] Bhattacharjee S., Bhattacharjee A., Majumder S., Majumdar S. B., Majumdar S. (2012). Glycyrrhizic
Acid Suppresses Cox-2-Mediated Anti-Inflammatory Responses during
Leishmania Donovani Infection. J. Antimicrob.
Chemother..

[82] Leroux M., Messaoud H. B.-B., Luquain-Costaz C., Jordheim L. P., Le Faouder P., Gustin M. P., Aoun K., Lawton P., Azzouz-Maache S., Delton I. (2023). Enriched PUFA Environment of Leishmania Infantum Promastigotes
Promotes the Accumulation of Lipid Mediators and Favors Parasite Infectivity
towards J774 Murine Macrophages. Lipids.

[83] Saini S., Kottarath S. K., Dinda A. K., Dube A., Sahasrabuddhe A. A., Thakur C. P., Bhat M., Rai A. K. (2020). Preventive as Well
as Therapeutic Significances of Linoleic Acid in the Containment of
Leishmania Donovani Infection. Biochimie.

[84] Lewis S. M., Williams A., Eisenbarth S. C. (2019). Structure-Function
of the Immune
System in the Spleen. Sci. Immunol.

[85] Venturin G. L., Bragato J. P., Melo L. M., Rebech G. T., Costa S. F., de Siqueira C. E., dos Santos Maciel M., de Rezende Eugênio F., Santos P. S. P., de Lima V. M. F. (2020). Regulatory Effect of PGE2 on Microbicidal
Activity and Inflammatory Cytokines in Canine Leishmaniasis. Parasite Immunol..

[86] Reiner N. E., Malemud C. J. (1984). Arachidonic Acid
Metabolism in Murine Leishmaniasis
(Donovani): Ex-Vivo Evidence for Increased Cyclooxygenase and 5-Lipoxygenase
Activity in Spleen Cells. Cell. Immunol..

[87] Pérez-Santos J. L. M., Talamás-Rohana P. (2001). In Vitro Indomethacin Administration
Upregulates Interleukin-12 Production and Polarizes the Immune Response
towards a Th1 Type in Susceptible BALB/c Mice Infected with Leishmania
Mexicana. Parasite Immunol..

[88] Soares M. B. P., David J. R., Titus R. G. (1997). An in Vitro
Model for Infection with
Leishmania Major That Mimics the Immune Response in Mice. Infect. Immun..

[89] Arcanjo A. F., LaRocque-de-Freitas I. F., Rocha J. D. B., Zamith D., Costa-da-Silva A. C., Nunes M. P., Mesquita-Santos F. P., Morrot A., Filardy A. A., Mariano M., Bandeira-Melo C., DosReis G. A., Decote-Ricardo D., Freire-de-Lima C. G. (2015). The PGE2/IL-10
Axis Determines Susceptibility of B-1 Cell-Derived Phagocytes (B-1CDP)
to Leishmania Major Infection. PLoS One.

[90] Mears E. R., Modabber F., Don R., Johnson G. E. (2015). A Review: The Current
In Vivo Models for the Discovery and Utility of New Anti-Leishmanial
Drugs Targeting Cutaneous Leishmaniasis. PLoS
Neglected Trop. Dis..

[91] van
der Ende J., Schallig H. D. F. H. (2023). Leishmania Animal Models Used in
Drug Discovery: A Systematic Review. Animals.

[92] Sacks D., Noben-Trauth N. (2002). The Immunology
of Susceptibility and Resistance to
Leishmania Major in Mice. Nat. Rev. Immunol..

[93] Nieto A., Domínguez-Bernal G., Orden J. A., De La
Fuente R., Madrid-Elena N., Carrión J. (2011). Mechanisms
of Resistance and Susceptibility to Experimental Visceral Leishmaniosis:
BALB/c Mouse versus Syrian Hamster Model. Vet.
Res..

[94] Kiser E. T., Wacker M. A., Dixit U. G., Batra-Sharma H., Chen Y., Wilson M. E. (2021). The Inflammatory Effects of Dietary
Lipids Regulate Growth of Parasites during Visceral Leishmaniasis. mSphere.

[95] Bandyopadhyay S., Bhattacharjee A., Banerjee S., Halder K., Das S., Chowdhury B. P., Majumdar S. (2015). Glycyrrhizic Acid-Mediated Subdual
of Myeloid-Derived Suppressor Cells Induces Antileishmanial Immune
Responses in a Susceptible Host. Infect. Immun..

[96] Volpedo G., Oljuskin T., Cox B., Mercado Y., Askwith C., Azodi N., Bernier M., Nakhasi H. L., Gannavaram S., Satoskar A. R. (2023). Leishmania Mexicana
Promotes Pain-Reducing Metabolomic
Reprogramming in Cutaneous Lesions. iScience.

[97] Tans R., Dey S., Dey N. S., Cao J. H., Paul P. S., Calder G., O’Toole P., Kaye P. M., Heeren R. M. A. (2022). Mass Spectrometry
Imaging Identifies Altered Hepatic Lipid Signatures during Experimental
Leishmania Donovani Infection. Front. Immunol..

[98] Yuan D., Qin H., Yu Z. (2025). Integration
of Hepatic Lipidomics and Transcriptomics
Reveals Dysregulation of Lipid Metabolism in a Golden Hamster Model
of Visceral Leishmaniasis. Front. Immunol..

[99] de
Sousa Andrade Y. M. F., Goicochea A. M. C., Jesus-Santos F. H., Fontes J. L. M., Mesquita B. R., deMelo C. V. B., Hlavac N., Bonyek-Silva I., da Silva Solcà M., Fraga D. B. M., Vilela A. F. L., Sorgi C. A. (2025). Arachidonic Acid Metabolism
and HETEs-PGs Imbalance. ACS Omega.

[100] França-Costa J., Andrade B. B., Khouri R., Van Weyenbergh J., Malta-Santos H., Da Silva Santos C., Brodyskn C. I., Costa J. M., Barral A., Bozza P. T., Boaventura V., Borges V. M. (2016). Differential Expression of the Eicosanoid
Pathway in
Patients with Localized or Mucosal Cutaneous Leishmaniasis. J. Infect. Dis..

[101] Malta-Santos H., Fukutani K. F., Sorgi C. A., Queiroz A. T. L., Nardini V., Silva J., Lago A., Carvalho L. P., Machado P. L. R., Bozza P. T., França-Costa J., Faccioli L. H., Carvalho E. M., Andrade B. B., Borges V. M. (2020). Multi-Omic
Analyses of Plasma Cytokines, Lipidomics, and Transcriptomics Distinguish
Treatment Outcomes in Cutaneous Leishmaniasis. iScience.

[102] Malta-Santos H., Andrade B. B., Zanette D. L., Costa J. M., Bozza P. T., Bandeira-Melo C., Barral A., Francą-Costa J., Borges V. M. (2017). Resolvin D1 Drives Establishment of Leishmania Amazonensis
Infection. Sci. Rep..

[103] Araújo-Santos T., Andrade B. B., Gil-Santana L., Luz N. F., Dos Santos P. L., De Oliveira F. A., Almeida M. L., De Santana Campos R.
N., Bozza P. T., Almeida R. P., Borges V. M. (2017). Anti-Parasite Therapy Drives Changes
in Human Visceral Leishmaniasis-Associated Inflammatory Balance. Sci. Rep..

[104] Solcà M. S., Andrade B. B., Abbehusen M. M. C., Teixeira C. R., Khouri R., Valenzuela J. G., Kamhawi S., Bozza P. T., Fraga D. B. M., Borges V. M., Veras P. S. T., Brodskyn C. I. (2016). Circulating Biomarkers of Immune
Activation, Oxidative Stress and Inflammation Characterize Severe
Canine Visceral Leishmaniasis. Sci. Rep..

